# An ultrastructural investigation of tumors undergoing regression mediated by immunotherapy

**DOI:** 10.18632/oncotarget.23215

**Published:** 2017-12-14

**Authors:** Jennifer A. Westwood, Sarah Ellis, Jill Danne, Chad Johnson, Viola Oorschot, Georg Ramm, David C. Tscharke, Alexander J. Davenport, James C. Whisstock, Phillip K. Darcy, Michael H. Kershaw, Clare Y. Slaney

**Affiliations:** ^1^ Cancer Immunology Program, Peter MacCallum Cancer Center, Melbourne, Australia; ^2^ Sir Peter MacCallum Department of Oncology, University of Melbourne, Parkville, Australia; ^3^ Monash Ramaciotti Centre for Cryo Electron Microscopy, Monash University, Clayton, Australia; ^4^ John Curtin School of Medical Research, Australian National University, Canberra, Australia; ^5^ The ARC Centre of Excellence in Advanced Molecular Imaging, Monash University, Melbourne, Australia

**Keywords:** electron microscopy, tumor regression, cancer vaccine, breast cancer, apoptosis

## Abstract

While immunotherapy employing chimeric antigen receptor (CAR) T cells can be effective against a variety of tumor types, little is known about what happens within the tumor at an ultrastructural level during tumor regression. Here, we used transmission electron microscopy to investigate morphologic and cellular features of tumors responding to immunotherapy composed of adoptive transfer of dual-specific CAR T cells and a vaccine, supported by preconditioning irradiation and interleukin-2. Tumors responded rapidly, and large areas of cell death were apparent by 4 days after treatment. The pleomorphic and metabolically active nature of tumor cells and phagocytic activity of macrophages were apparent in electron microscopic images of tumors prior to treatment. Following treatment, morphologic features of various types of tumor cell death were observed, including apoptosis, paraptosis and necrosis. Large numbers of lipid droplets were evident in tumor cells undergoing apoptosis. Macrophages were the predominant leukocyte type infiltrating tumors before treatment. Macrophages decreased in frequency and number after treatment, whereas an increasing accumulation of neutrophils and T lymphocytes was observed following treatment. Phagocytic activity of macrophages and neutrophils was apparent, while T cells could be observed in close association with tumor cells with potential immunological synapses present. These observations highlight the cellular composition and ultrastructural appearance of tumors undergoing regression mediated by immunotherapy.

## INTRODUCTION

Immunotherapy can be highly effective against cancer. Some leukemias and other hematologic cancers can be eradicated using adoptive cell transfer (ACT) in a proportion of individuals who have failed all standard treatments [1–3]. Large burdens of metastatic melanoma can also respond completely to immune-based treatments, particularly those using ACT [4–8].

The use of monoclonal antibodies to block immunosuppressive checkpoint receptors is also achieving remarkable success in the treatment of some malignancies including melanoma and cancers of the lung and kidney [9, 10]. Furthermore, associations between the magnitude of lymphocyte infiltrate in tumors and patient outcome have been demonstrated in solid tumors other than melanoma, including cancers of the colon and ovaries [11–13]. suggesting that immunotherapy may one day be effective against a large range of common malignancies.

Mechanistically, a variety of leukocytes and molecules have been identified as important in mediating anti-tumor responses following immunotherapy. For example, T cells [14], NK cells [15] and macrophages [16] have been demonstrated to exert anti-tumor action. In addition, some leukocytes can play a negative role by suppressing immune responses and promoting tumor growth [17].

Visually, elegant studies using live cell microscopy *in vitro* have demonstrated the ability of CAR T cells to serially kill several cancer cells [18]. In addition, ground-breaking studies using intravital microscopy have characterized the distribution and movement of various leukocytes in tumors following immunotherapy [19–21].

In recent work, we described a combination immunotherapy approach using adoptive cell transfer incorporating vaccination (ACTIV) [22]. ACTIV therapy comprised a combination of a preconditioning lymphodepleting irradiation (5 Gy), followed by adoptive transfer of dual-specific T cells reactive with Her2, through a chimeric antigen receptor (CAR) and able to respond through their T cell receptor (TCR) to a melanocyte antigen, pMEL (gp100). ACT was followed by intravenous injection of vaccinia virus encoding gp100, and intraperitoneal delivery of IL-2. We demonstrated that ACTIV therapy could eradicate established solid tumors, from several different histological origins, in a syngeneic self-antigen setting in mice. The study included the orthotopic breast cancer model using E0771-Her2 cells, which showed that large tumors, some in excess of 150 mm^2^, could be eradicated.

Nevertheless, despite these demonstrations of the successes of cancer immunotherapy and elegant mechanistic studies, there is a paucity of information about the ultrastructural appearance of tumors while in the process of immune mediated remission. In this study, we present a phenotypic and ultrastructural characterization of tumors undergoing a massive, often complete response to immunotherapy.

## RESULTS

### ACTIV therapy induces widespread regions of tumor cell death and extensive immune cell infiltration

**Figure 1:**
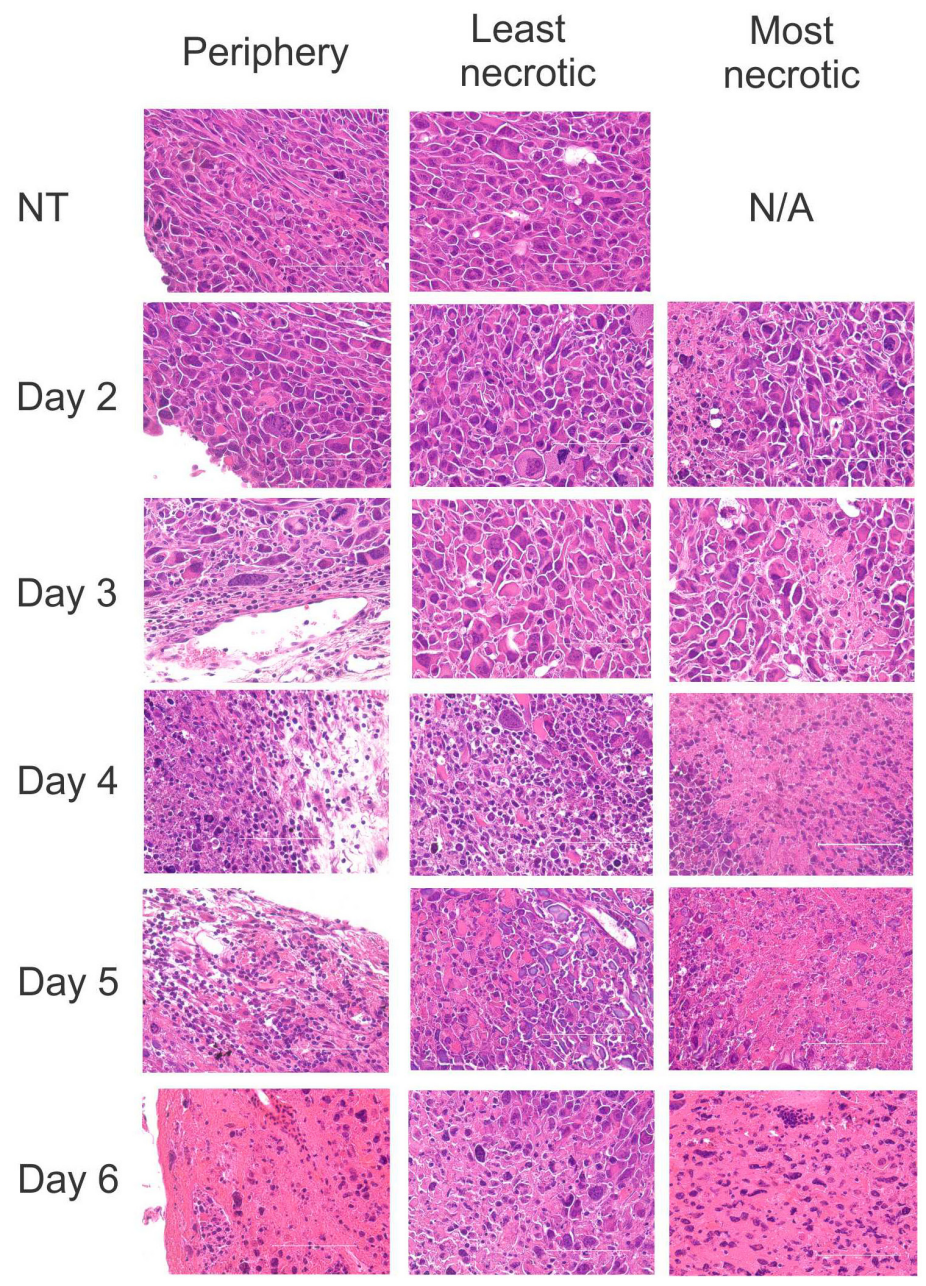
ACTIV therapy induces tumor cell death and leukocyte infiltration Mice were injected subcutaneously with E0771-Her2 cells and tumors allowed to grow for 14 days, at which time they were approximately 50-70 mm^2^ in size. Mice were then either left untreated or received ACTIV therapy. Tumors were taken from cohorts of mice at various time points after therapy, as listed, and H&E-stained sections prepared. Representative fields from multiple sections of three tumors of the tumor periphery are presented, together with areas representative of the least necrotic or most necrotic regions. Arrows indicate examples of infiltrating leukocytes. Scale bar = 100 μm.

In previous experiments, ACTIV treatment of E0771-Her2 tumors in mice typically led to an approximate reduction in tumor size of 50% by day 5 after treatment, and complete tumor regression by Day 20. To gain insight into changes to tumors following therapy, histologic analysis was performed on tumors taken at various time points from ACTIV-treated or non-treated mice. Following treatment, scattered regions of necrosis became visible in tumors as early as Day 2, which progressively increased in extent by Day 6 (Figure 1). A pronounced leukocyte infiltrate was visible in the periphery of tumors by Day 3, which extended into the core of tumors by Days 4 – 6. The most florid leukocyte infiltrate was associated with necrotic regions, suggesting that immune cells were responsible for tumor cell destruction and/or were attracted to regions of tissue degradation.

### The immune cell composition of tumors varies with time after ACTIV therapy

**Figure 2:**
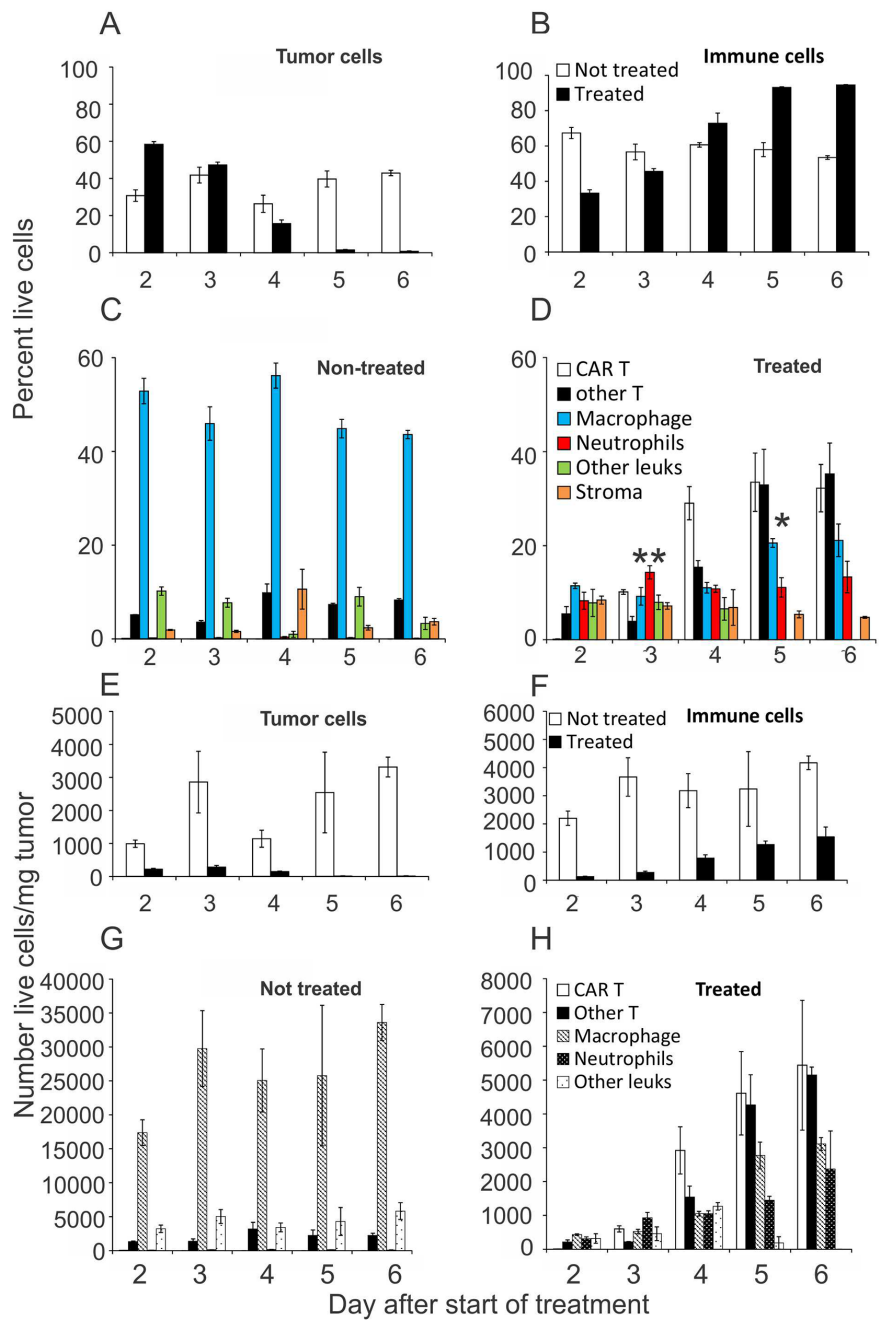
Quantitation of cellularity of tumors following ACTIV therapy Mice were injected subcutaneously with E0771-Her2 cells and ACTIV therapy administered 14 days later. Tumors were taken from treated or non-treated cohorts of mice at the listed time points after therapy and were enzymically dissociated, stained with specific antibodies and analysed using flow cytometry. **(A, B)** Frequency of tumor cells or leukocytes in tumors on the indicated days after the start of treatment. **(C, D)** Frequency of leukocyte subsets infiltrating treated or non-treated tumors. **(E-H)** Numbers/mg of tumor cells or leukocytes, in either treated or non-treated tumors, as listed. Values represent the average (n = 3), and error bars represent the SEM. ^**^ p=0.0006 frequency of neutrophils in tumors 3 days after treatment compared to tumors receiving no treatment. ^*^ p=0.0011 frequency of macrophages in treated tumors on Day after treatment compared to Day 2 (unpaired 2-tail Student *T* test).

Tumors were taken from non-treated mice, or ACTIV-treated mice at time intervals following treatment, and subjected to enzymic dissociation and flow cytometric analysis. When the cellular composition of dissociated tumors were expressed as percentage of live cells, the proportion of tumor cells in non-treated tumors stayed relatively similar over time at approximately 30% - 40% (Figure 2A). In contrast, in ACTIV-treated tumors the proportion of tumor cells decreased from >50% on Day 2 to <5% on Day 6, indicating rapid and extensive elimination of the majority of live tumor cells. Correspondingly, while the percentage of infiltrating immune cells in non-treated tumors remained constant at 50% - 70%, immune cells in treated tumors increased in proportion towards approximately 95% by Day 5 (Figure 2B), suggesting either preferential survival of leukocytes and/or their active recruitment to tumors.

Phenotypic analysis of infiltrating cells revealed that the proportions of leukocyte types remained relatively constant in non-treated tumors, with macrophages comprising the majority of the tissue (Figure 2C). Conversely, in treated tumors, the proportions of leukocyte subsets changed over time (Figure 2D). CAR T cells appeared by Day 3 and their frequency increased to approximately 30% by Days 5 and 6, suggesting an active attack on tumors. This increased accumulation of CAR T cells (following adoptive transfer) was also accompanied by a similar increase in infiltration by endogenous T cells. In addition, a significant increase in the percentage of neutrophils was observed by Day 3 (P = 0.0006, Unpaired 2-tailed Student *T* test), and macrophages progressively increased in frequency from approximately 10% on Day 2 to 20% by Day 5 (P = 0.0011). Thus, a dynamic process of tissue remodelling and cellular repopulation occurred following treatment that was associated with tumor destruction and healing of lesions. Alternatively, since this part of the analysis only involved determining the frequency of cells, it is possible that some cell types were preferentially retained and proliferated in situ, rather than newly recruited.

Interestingly, when the cellular composition of dissociated tumors was expressed as the actual number of live cells per milligram of tumor, the effect of treatment was even more dramatic than that suggested by the analysis of percentages. A large reduction in the number of live tumor cells was observed following treatment, with an approximately 80% reduction in live tumor cells as early as Day 2 and further reductions in subsequent days until only several hundred live tumor cells per milligram could be found (Figure 2E).

The impact of treatment on the immune compartment of tumors was also profound, with a >90% reduction in numbers compared to non-treated tumors (Figure 2F). This was likely due to the preconditioning regimen of 5 Gy irradiation, designed to lymphodeplete tumor hosts to enable engraftment of transferred T cells. The numbers of individual immune cell subsets was also significantly affected by treatment.

Whereas the numbers of macrophages in non-treated tumors remained relatively stable at approximately 20,000 – 30,000 cells/mg (Figure 2G), their numbers in treated tumors were reduced to < 1,000 cells/mg on Day 2 and only recovered to approximately 3,000 cells/mg by Day 6 (Figure 2H).

In contrast, neutrophils were relatively rare in non-treated tumors (∼100 cells/mg), but increased in treated tumors to reach in excess of 10% of live cells (1000 cells/mg) by Day 6 (Figure 2H). The increasing numbers of phagocytes over the treatment period likely indicated mediation of tissue repair in response to treatment-induced damage to tumors, although we cannot rule out a role for phagocytes in removal of malignant cells.

Regarding T cells, the initial decrease in their numbers in tumors, expected after irradiation, was followed by their rapid accumulation in tumors by Day 5 to exceed the numbers in non-treated tumors by approximately 3-fold (Figure 2G, 2H). The T cell population was composed of approximately equal proportions of CAR T cells and endogenous T cells, suggesting T cell recruitment to tumors was largely non-specific.

Interestingly, when the yield of total cells in suspensions derived by enzymic digestion was compared to numbers calculated by counting hematoxylin/eosin-stained slides, it was apparent that there was a large discrepancy between the two methods. Enumeration of total cells by microscopy estimated approximately 400,000 cells/mg in non-treated tumors, but the total yield from dissociation was typically less than 80,000, indicating a loss of 80% of cells. This loss could have been from cell death during processing and/or incomplete release of cells from the tumor. The above data concerning cell frequencies and numbers therefore need to be taken in context of this cell loss, and disproportionate loss of individual cell types needs to be considered when interpreting data derived from flow cytometry of dissociated tumors.

**Figure 3:**
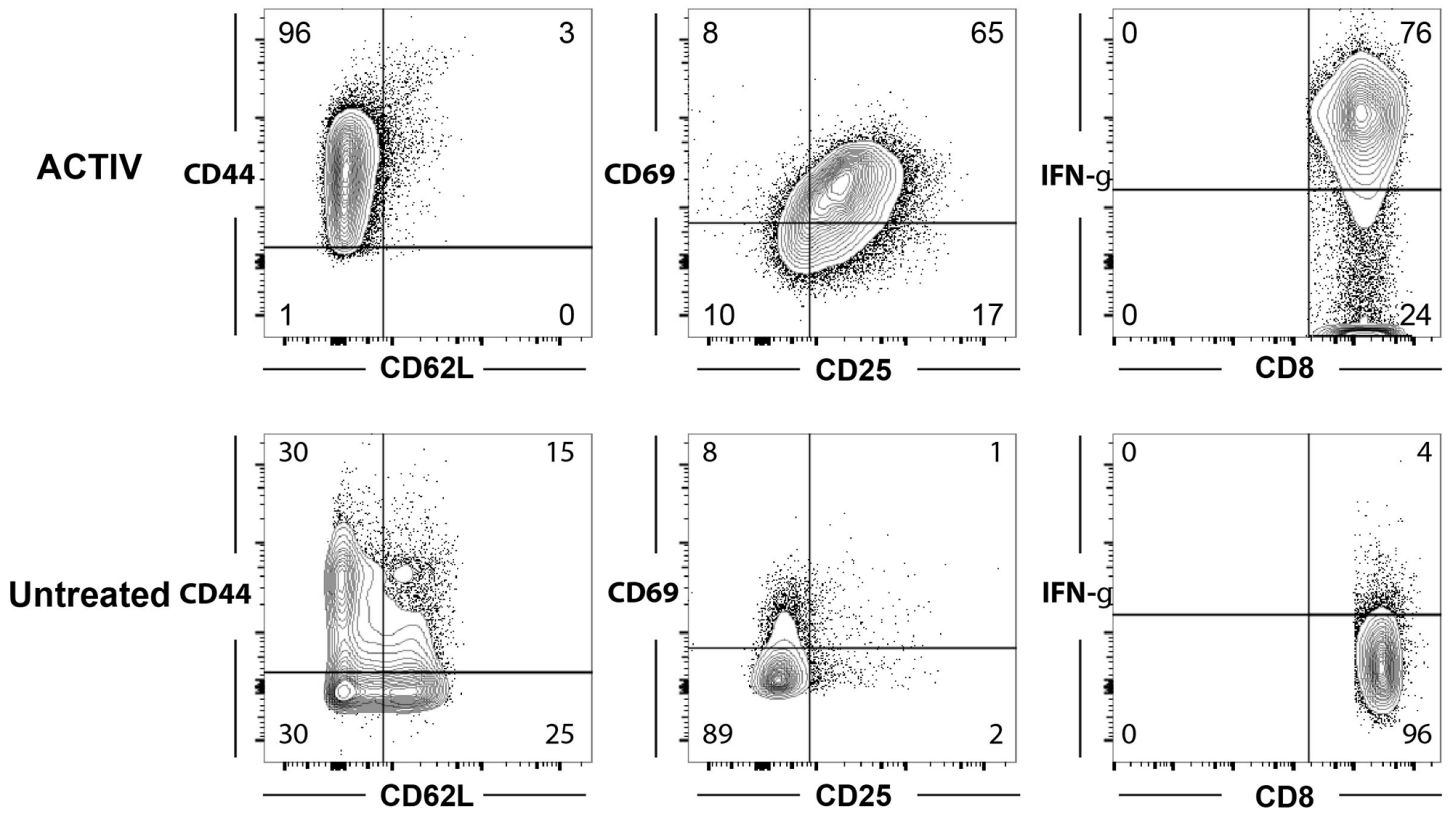
Tumor-infiltrating T cells in ACTIV-treated mice have an activated phenotype E0771-Her2 tumors were taken from non-treated mice (lower panels) or mice 6 days after ACTIV treatment (upper panels) and enzymically dissociated. Tumor samples were then stained with monoclonal antibodies specific for the markers listed, and analysis by flow cytometry after gating on CD8^+^ T cells. This data is representative of two experiments.

To further characterize the T cells within tumors, we analysed their expression of activation markers (CD44, CD62L, CD69 and CD25) and functional marker (intracellular IFN-γ). We focused on CD8^+^ T cells, since these had been identified as necessary and sufficient for therapy in our previous study [22]. Interestingly, CD8^+^ T cells in treated mice were largely CD44^high^, CD62L^low^, CD69^+^, CD25^+^ and expressed high amounts of IFN-γ, indicating an effector phenotype, whereas CD8^+^ T cells in non-treated mice were a mixture of effector, effector memory, central memory and naïve phenotypes (Figure 3).

### Electron microscopy reveals the pleomorphic nature of cancer cells and infiltrating macrophages

**Figure 4:**
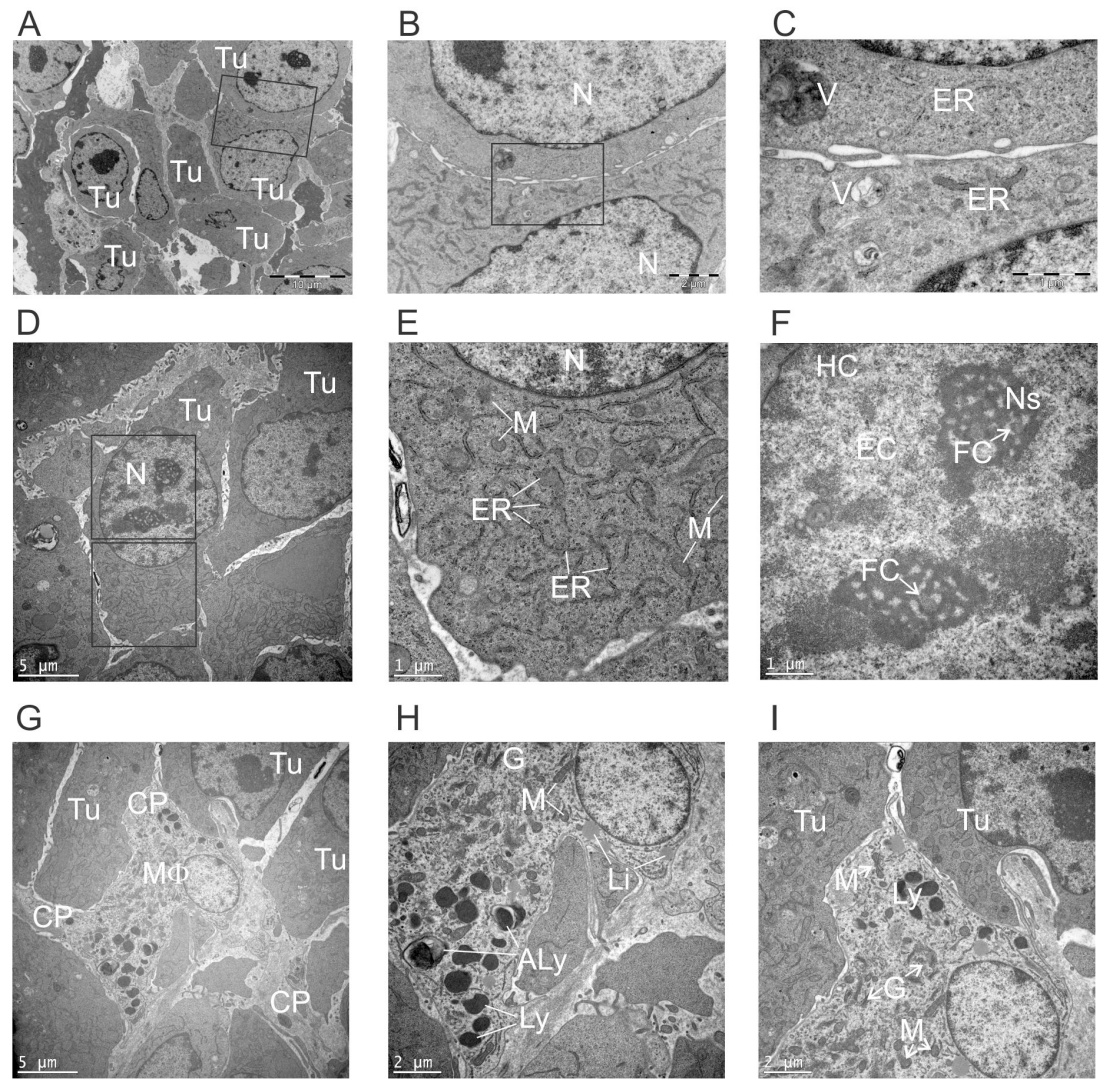
Ultrastructural appearance of non-treated tumors Subcutaneous E0771-Her2 tumors were taken 14 days after injection, and analysed using transmission electron microscopy. Representative fields depicting tumor cells (**A-F**) and macrophages (**G-I**) are presented. (B) High power view of boxed region in A. (C) High power view of boxed region in B. (E, F) Higher power view of boxed regions in D. (Tu, tumor cell; N, nucleus; ER, endoplasmic reticulum; V, vesicle; M, mitochondria; HC, heterochromatic; EC, euchromatin; FC, fibrillary centers; Ns, nucleolus; MF, macrophage; CP, cytoplasmic process; G, golgi; Ly, lysosome; Aly, autolysosome; Li, lipid droplet.

Non-treated tumors were largely composed of densely packed tumor cells interspersed with macrophages. Tumor cells were pleomorphic without apparent glandular arrangement or polarity (Figure 4A, 4D). The cytoplasm included occasional vacuoles, large amounts of endoplasmic reticulum (ER) and numerous scattered mitochondria, generally round in shape, suggestive of metabolically active cells (Figure 4B, 4C, 4E). Tumor cell nuclei were round in shape and comprised largely of euchromatin with one or two nucleoli, reminiscent of actively growing cells. Multiple fibrillary centers were visible in nucleoli, suggesting highly active manufacture of ribosomal components (Figure 4F).

Macrophages could be seen infiltrating tumor tissue, often with cytoplasmic processes and an elongated shape suggestive of M2 macrophage migratory activity (Figure 4G) [23]. Mitochondria were frequent, often with a classic peanut shape (Figure 4H, 4I). The high frequency of mitochondria suggested metabolically active cells using oxidative phosphorylation, which is associated with M2 macrophages that are often involved in immunosuppression and tissue repair [24]. Golgi were frequently present, suggesting secretory activity (Figure 4H, 4I). Lysosomes could also be seen, indicating phagocytic activity. Interestingly, autolysosomes were sometimes present, suggesting some degree of cellular stress experienced by tumor-infiltrating macrophages. However, it is important to note that the above observations are largely suggestive based on structural findings, and supporting evidence from immunological or biochemical measurements in future studies would be needed before firm conclusions about macrophage function could be made.

### ACTIV-treated tumors undergo massive progressive ultrastructural changes

**Figure 5:**
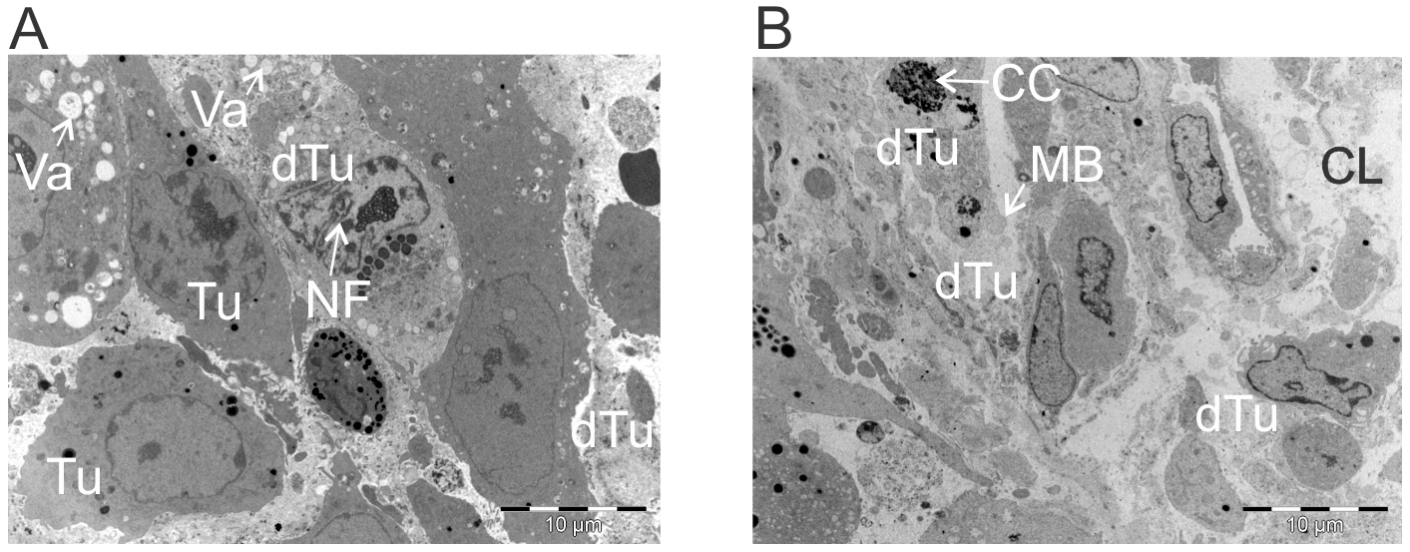
ACTIV-treated tumors display morphologic characteristics of apoptosis, paraptosis and necrosis E0771-Her2 tumors were taken 4 days after ACTIV treatment and analysed using transmission electron microscopy. **(A)** Representative image of a region of low-level cell death. **(B)** Representative image of an area of high-level cell death. Tu, live tumor cell; dTu, dying tumor cell; Va, vacuole; NF, nuclear fragmentation; CC, chromatin condensation; MB, membrane blebbing; CL, complete lysis.

Significant reduction in tumor size was observed by Day 4 following ACTIV therapy. Using electron microscopy at this time point, tumor cells at various stages of death and dissociation could be seen. Some regions of tumors exhibited low levels of tumor death (Figure 5A), whereas more extensive regions of death were present in other regions (Figure 5B). Features of apoptosis, paraptosis and necrosis [25] could be seen. Cytoplasmic vacuolation, characteristic of paraptosis was observed in some tumor cells, and nuclear fragmentation, characteristic of apoptosis was also present (Figure 5A). Chromatin condensation and membrane blebbing, associated with apoptosis, was also observed in some areas, whereas complete loss of cytoplasmic integrity and lysis was seen in other regions (Figure 5B). The ultrastructural appearance of apoptotic tumor cell death supported previous findings of extensive apoptosis in ACTIV-treated tumors, demonstrated by the presence of cleaved caspase-3 [22].

**Figure 6:**
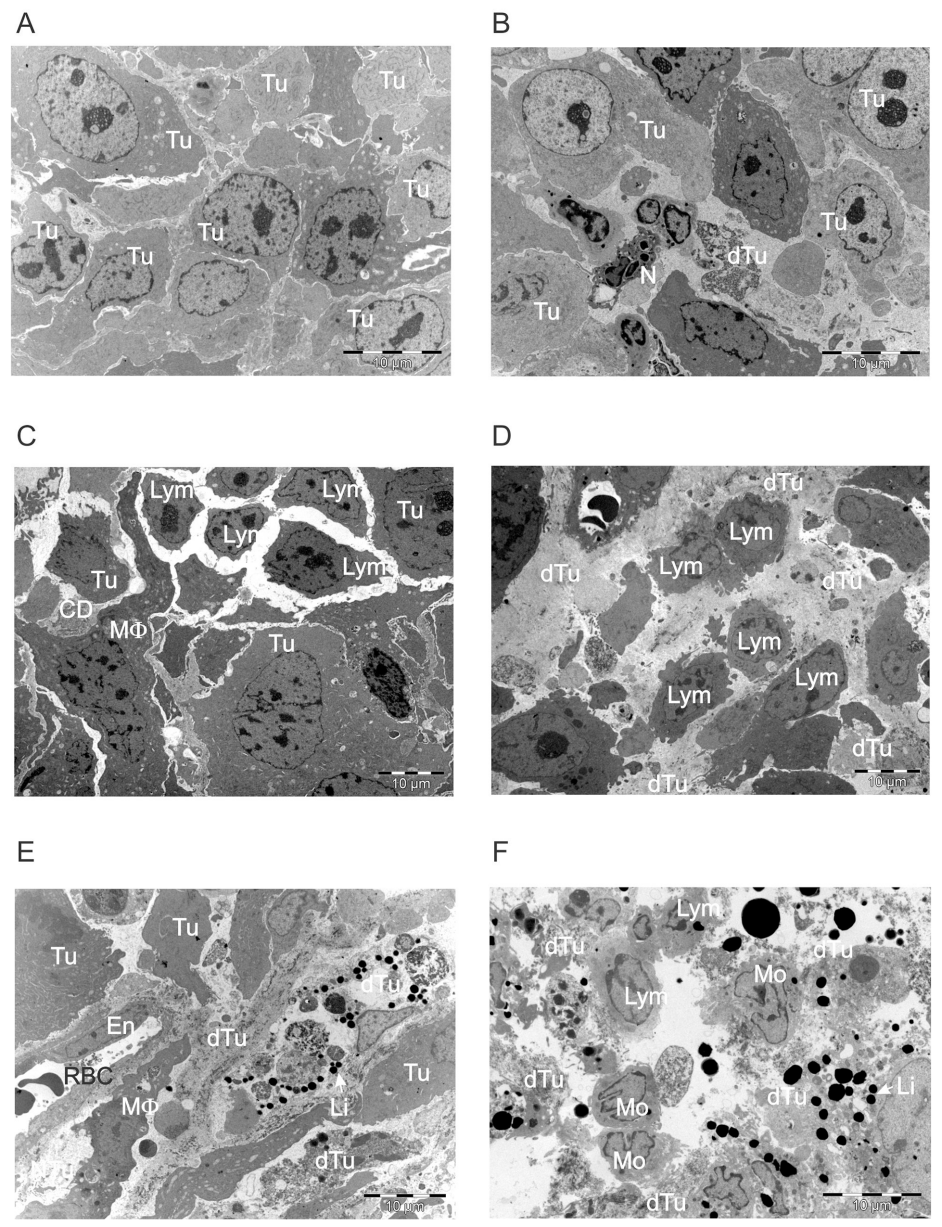
ACTIV therapy induces progressive tumor cell death and increased leukocyte infiltration over time Established subcutaneous E0771-Her2 tumors were treated with ACTIV therapy and taken at the various time points listed, and analyzed using transmission electron microscopy. **(A)** Non-treated tumor, **(B-F)** tumors at Days 2-6, respectively, after treatment. Tu, live tumor cell; dTu, dying tumor cell; N, neutrophil; Lym, lymphocyte; CD, cell debris; MF, macrophage; En, endothelial cell; RBC, red blood cell; Mo, mononuclear cell, likely monocyte or lymphocyte; Li, lipid droplet.

Analysis of tumor sections at various time points after ACTIV treatment showed the development of large areas of tumor cell death and the progressive infiltration of leukocytes (Figure 6). Before treatment, tumors were largely composed of closely packed cancer cells (Figure 6A), but changes were observed by Day 2 after treatment, with more frequent dead tumor cells and some infiltrating neutrophils and other leukocytes present (Figure 6B). The frequency of tumor-infiltrating leukocytes increased in subsequent days, with lymphocytes predominating (Figure 6C, 6D). Lymphocytes had a relatively large nucleus:cytoplasm ratio and an irregular cytoplasmic membrane, characteristic of activation and migratory activity. The lymphocytes included those derived from the adoptively transferred CD8^+^ dual-specific T cells, since this was previously described as a major tumor-infiltrating leukocyte using immunohistochemistry and flow cytometry [22].

**Figure 7:**
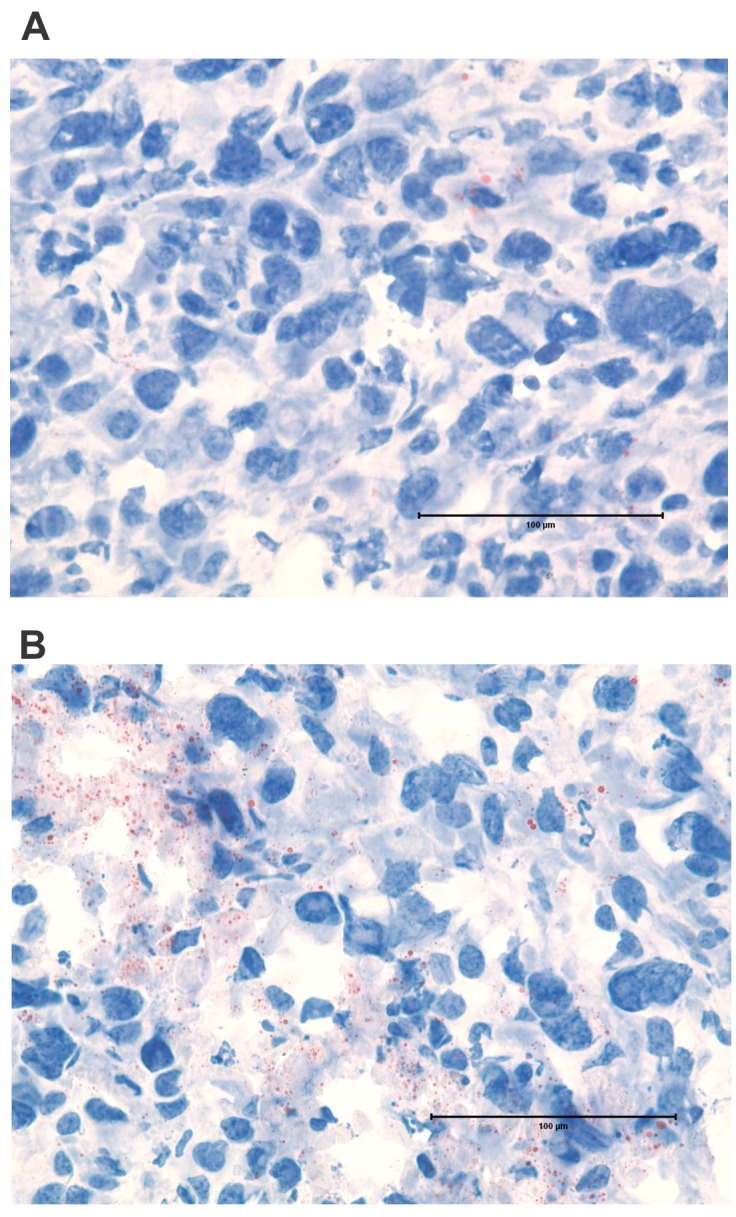
Lipid droplets form in tumor cells following ACTIV therapy Sections of subcutaneous E0771-Her2 tumors were stained with Oil Red O and analyzed using light microscopy. Sections shown are representative of **(A)** non-treated tumors and **(B)** tumors 5 days after ACTIV treatment. Lipid droplets stain red. Size bar = 100 μm.

Regions of dead tumor cells increased over time after treatment, until large areas of ultrastructural devastation were evident by Days 5 and 6 (Figure 6E, 6F). The appearance of electron dense bodies was noted by Days 4-6, which were initially present in the cytoplasm of tumor cells, but could subsequently be seen scattered throughout areas of necrosis as tumor cells disintegrated. The morphology of these electron dense bodies and their association with apoptotic tumor cells suggested they were lipid droplets (Figure 6E, 6F), since lipid droplet formation has previously been demonstrated to be associated with apoptosis [26–28]. The lipid nature of the electron dense bodies was supported by staining of tumor sections using Oil Red O, which highlighted lipid droplets of similar morphology and distribution as that of the electron dense bodies (Figure 7).

### The ultrastructural appearance of leukocytes infiltrating tumors following ACTIV therapy

**Figure 8:**
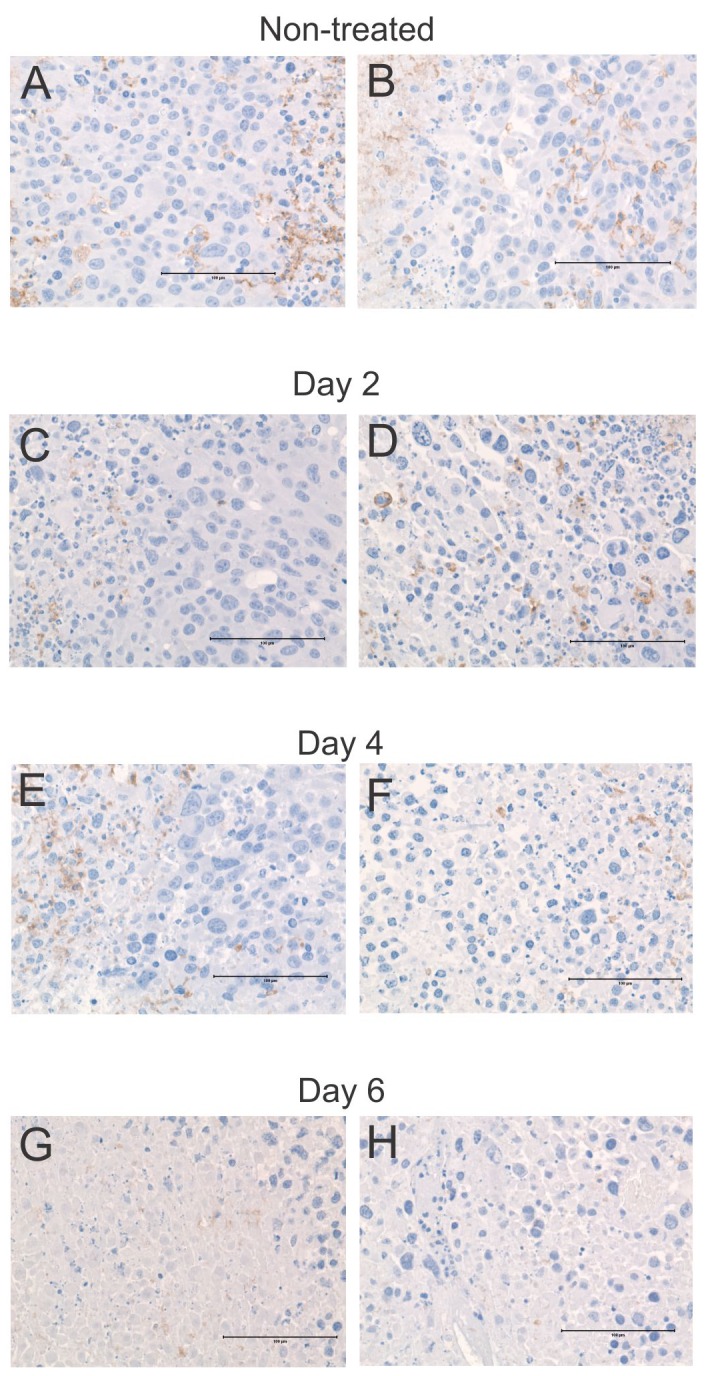
Frequency and distribution of macrophages in tumors following ACTIV therapy Representative sections of tumors were taken from mice following **(A-B)** no treatment, **(C-D)** 2 days after treatment, **(E-F)** 4 days after treatment, **(G-H)** 6 days after treatment, and stained with an antibody specific for the macrophage marker F4/80. Brown stain = F4/80, blue = hematoxylin. Size bar = 100 μm.

Having demonstrated that a variety of leukocyte types infiltrate tumors following ACTIV therapy (Figure 2), we sought insight into their distribution and morphology using immunohistochemistry and transmission electron microscopy. When sections of non-treated tumors were stained with the macrophage marker, F4/80, and examined using light microscopy, cells were observed with typical macrophage morphologic features including a relatively large and irregular shaped cytoplasm. Their distribution in non-treated tumors was usually not uniform throughout tumor tissue, but rather was more focal in nature and tended to be associated with necrotic regions (Figure 8A, 8B). This pattern of distribution continued after treatment, but the number of F4/80^+^ macrophages per field was less than in non-treated tumors (Figure 8C-8H). Macrophages were not evenly distributed throughout treated tumors, rather they were usually located in focused regions associated with dying or dead tumor tissue.

**Figure 9:**
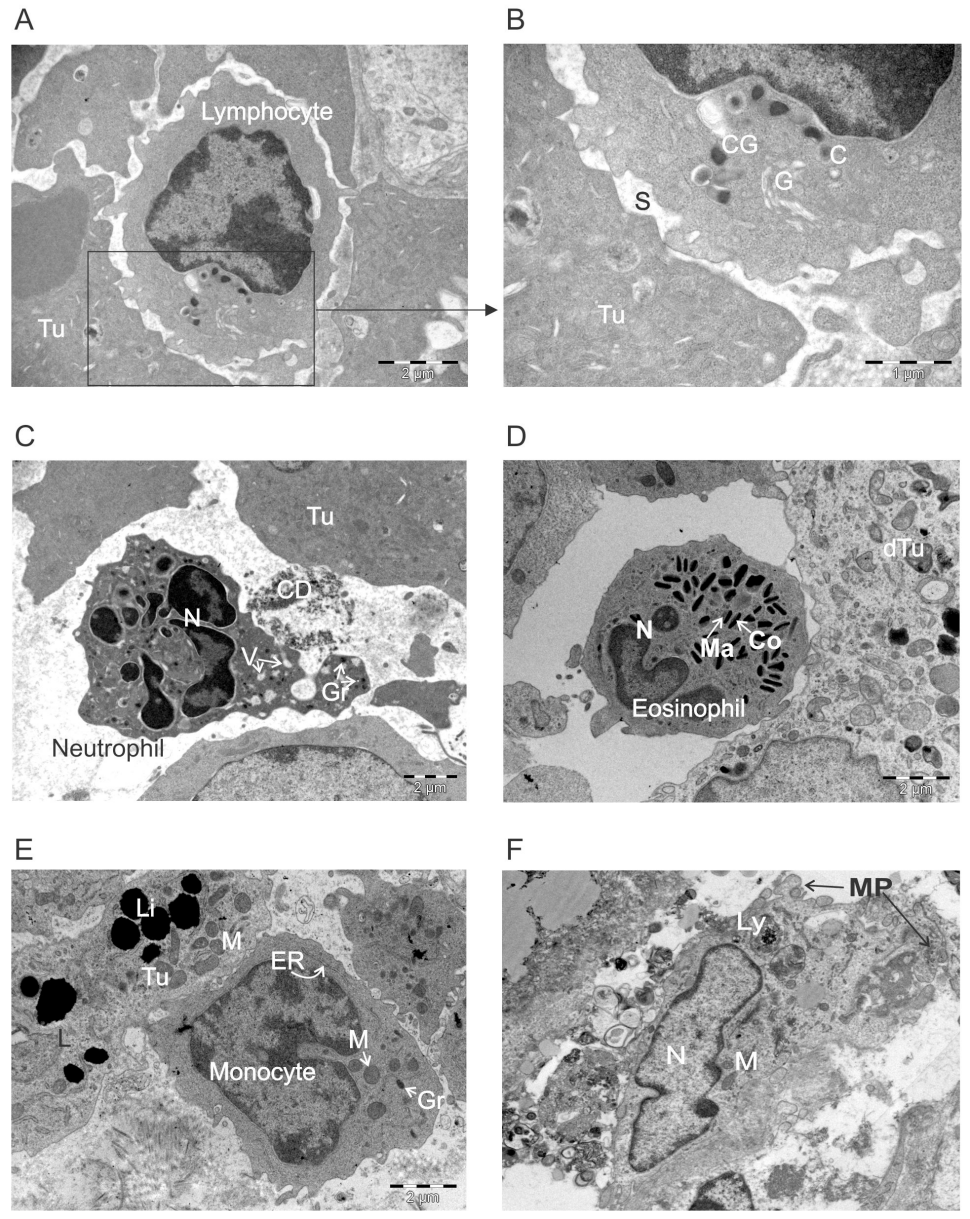
Ultrastructural appearance of leukocytes infiltrating ACTIV-treated tumors E0771-Her2 tumors were taken on day 5-6 after ACTIV treatment and analyzed using transmission electron microscopy. Representative images of immune infiltrating cells are presented depicting: **(A-B)** a lymphocyte interacting with a tumor cell, **(C)** neutrophil, **(D)** eosinophil, **(E)** monocyte, **(F)** macrophage. Abbreviations: Tu, live tumor cell; C, centriole; CG, cytotoxic granule; G, golgi; S, synapse; CD, cell debris; Gr, granule; V, vacuole; N, nucleus; Ma, matrix; Co, core; dTu, dying tumor cell; ER, endoplasmic reticulum; M, mitochondria; Li, lipid droplets; Ly, lysosome; MP, membrane processes.

The most frequently observed leukocytes within tumors on Day 6 after ACTIV therapy were lymphocytes. Some lymphocytes were in contact with tumor cells and, although no specific staining was done, the high frequency of adoptively transferred tumor-reactive T cells (Figure 2D), suggested that a large proportion of lymphocytes observed using electron microscopy were T cells, and that their contact with tumor cells was a specific response against tumor. Indeed, some of the lymphocyte-tumor interactions appeared to involve a synapse-like structure with focal adhesion points, and with centrosome, golgi and cytoplasmic granules within lymphocytes orientated towards the synapse (Figure 9A, 9B).

Although not as frequent as lymphocytes, neutrophils were often observed throughout ACTIV-treated tumors, and exhibited the typical multi-lobed nucleus, together with multiple cytoplasmic vacuoles and granules. Some neutrophils seemed to possess membrane extensions wrapping around cellular debris, likely in the process of phagocytosis (Figure 9C).

Eosinophils were only occasionally found and their presence was likely coincidental rather than specific. Their characteristic lobed nucleus could be seen together with granules (Figure 9D). The electron-dense core of granules and less dense surrounding matrix could be clearly seen, and granules appeared to be full with no evidence of significant secretion.

Monocytes could also be found within treated tumors, which possessed a characteristic horseshoe-shaped appearance, in addition to few lysosomal granules (Figure 9E). The second most frequently observed leukocyte within treated tumors were macrophages, often found in areas of cell death and decomposition, where they were engaged in phagocytosis (Figure 9F). Membrane processes were often observed engaged in enclosure of cell debris, and their cytoplasm often contained large amounts of granular and lysosomal material, again reminiscent of phagocytosis and breakdown of engulfed material.

## DISCUSSION

In this study, we characterized the cellular composition of tumors undergoing rapid responses following immunotherapy. Transmission electron microscopy was used to gain insight into the distribution, morphology and ultrastructural appearance of cancer cells and infiltrating leukocytes during tumor regression.

The immunotherapy regimen consisted of preconditioning radiation followed by adoptive transfer of tumor-reactive dual-specific T cells, and a recombinant vaccinia virus vaccine together with administration of IL-2.

The traditional view of tumor responses to immunotherapy suggests that treatment leads to a slowing or halting of tumor growth followed by a gradual reduction in tumor viability and size. Indeed, our previous observations of tumor growth show growth retardation, followed by a slow decrease in size and resolution of disease [22]. However, from this study, a different picture emerges, which demonstrates that, rather than a gradual response process, a rapid induction of morphologic devastation occurs. By Day 6 after the start of treatment, despite the presence of a clearly measurable lump, there are huge areas of tumor death, with very few live tumor cells present.

The morphologic features of tumor cell death suggested that several forms of death were operating within tumors, including apoptosis, paraptosis and necrosis. This likely reflected varying mechanisms of cell death including direct effects of cytotoxicity from CTL as well as direct and indirect effects of radiation on tumor cells and stroma.

The most frequently observed leukocyte type in treated tumors were T lymphocytes, which were composed of both transferred tumor-specific T cells and endogenous T cells. Although the specificity of the endogenous T cells is not known, it seems unlikely they were all tumor-specific and so it seems T cell accumulation was potentially non-specific, perhaps due to the production of appropriate chemokines within tumors. The T cells possessed a relatively smaller nucleus:cytoplasm ratio compared to resting, naïve lymphocytes, suggesting the infiltrating T cells were of an activated phenotype. The presence of some cytoplasmic granules in some T cells also suggested they were activated, and likely CD8^+^ CTL. It would be of interest in future studies to perform immuno-EM studies to determine the origin and phenotype of infiltrating lymphocytes.

Macrophages and neutrophils were also frequently seen in TEM images, which were often involved in phagocytic activity. However, it is unclear at this stage whether they were also actively involved in anti-tumor activity, or simply participating in the removal of cell debris to effect resolution of damaged tissue.

Some other cells like eosinophils were also sometimes encountered. However, there was no evidence of participation in responses against tumor, such as through degranulation, and they may not have been engaged in specific responses, but they were likely just coincidental visitors.

Interestingly, electron-dense bodies were found associated with dead and dying tumor cells. The ultrastructure of these bodies was reminiscent of lipid droplets, but of a dense nature. We also considered the alternate interpretations of peroxisomes [29, 30] and condensed mitochondria [31], which can have a similar appearance, but Oil Red O staining indicated they were lipid droplets. The accumulation of lipid droplets likely indicates apoptosis of tumor cells and associated dysfunction of mitochondrial fatty acid metabolism leading to accumulation of lipids [26].

This study provides novel insight into tumor regression mediated by immunotherapy. Rapid and extensive destruction of tumors was observed that led to isolated individual tumor cells or isolated groups of tumor cells by day 6. Following on from our previous work demonstrating eradication of approximately 75% of tumors, the current study suggests that tumor relapse could be due to regrowth from rare isolated tumor cells. It is not known at present whether these isolated cells are inherently resistant to therapy or simply difficult for therapeutic elements to locate or access. This suggests that complete resolution of disease requires sufficient numbers of activated tumor-reactive leukocytes capable of searching out and destroying isolated pockets of tumor cells.

## MATERIALS AND METHODS

### Mice, cells and tumors

C57BL/6-Her2 mice expressed human Her2 under the control of the whey acidic protein (WAP) promoter [32]. C57BL/6-CARaMEL mice expressed a chimeric antigen receptor (CAR) specific for Her2 and a T cell receptor (TCR) specific for the melanocyte antigen, pMEL (gp100), as described previously [22, 33]. T cells derived from splenocytes of these mice were considered dual-specific, by virtue of expression of two antigen receptors, and were referred to as CARaMEL T cells. Mice were bred and maintained at the Peter MacCallum Cancer Center Animal Experimentation Facility.

The E0771-Her2 cell line, expressing human Her2 and the fluorescent marker, Cherry, was derived from a breast cancer cell line of C57BL/6 mice and maintained in DMEM as previously described [22].

Tumors were established in mice by injection of 2 × 10^5^ cells subcutaneously and allowed to grow for approximately 14 days, at which time they were either left untreated or received adoptive cell transfer incorporating vaccination (ACTIV) therapy. ACTIV therapy consisted of a preconditioning regimen of 5 Gy irradiation followed by intravenous transfer of 1 × 10^7^ splenocytes from CARaMEL mice, which typically contained approximately 2 × 10^6^ T cells. Tumor-bearing mice then received an intravenous injection of recombinant vaccinia virus expressing gp100 (VV-gp100, 2 × 10^7^ pfu) two hours after splenocyte administration, followed by 4 intraperitoneal injections of 2 × 10^5^ IU of human recombinant IL-2, spaced approximately 12 hours apart. Mice were utilized according to the requirements of the Peter MacCallum Cancer Center Animal Experimentation Ethics Committee.

### Flow cytometry

Tumors were dissected away from surrounding tissue, finely minced using scissors and incubated for 30 minutes at 37°C in DMEM containing collagenase IV (1 mg/ml, Worthington Biochemical, Lakewood, NJ) and DNAse I (30 units/ml, Sigma, Sydney, Australia). After washing in PBS, cells were stained with antibodies specific for TCR-β-PerCp5.5 (clone H57-597, eBioscience 45-5961-82), F4/80-BV421 (clone T45-2342, BD 565411) or CD11b-BV605 (clone M1/70, BD 563015) to detect T cells, macrophages or neutrophils respectively. CAR T cells were detected using the congenic marker Thy 1.1-APC (clone OX-7, BD 561409). Tumor cells were identified through expression of Cherry. Statistical significance was determined using a non-paired two-tail Student *T* test.

### Microscopy studies

#### Immunohistochemistry

Formalin fixed paraffin embedded sections 4 μm thick were dewaxed in histolene and rehydrated. A heat mediated antigen retrieval step was performed in sodium citrate buffer (pH6). Free aldehydes were blocked with glycine and endogenous peroxidase quenched with 3% hydrogen peroxidase. Non-specific binding was blocked using TNB buffer (0.1 M Tris-HCl, pH 7.5, 0.15 M NaCl, 0.5% (w/v) blocking reagent, PerkinElmer FP1020). Sections were incubated with rat anti-mouse F4/80 (Clone BM8, eBioscience 13-4801) or a Rat IgG2a isotype control (BD Pharmingen 553927) at a dilution of 5 μg/ml. To visualise the distribution of F4/80, sections were incubated with ImmPRESSTM HRP anti-rat IgG (Vector Laboratories MP-7444) and 3,3′-diaminobenzidine (DAKO K3468) according to the manufacturer’s instructions. Sections were counterstained with haematoxylin before coverslipping.

#### Oil Red O staining

Cryosections 5 μm thick were fixed in formalin, rinsed in 60% Isopropyl alcohol and stained with Oil Red O working solution as per standard histological methods. Following differentiation in 60% Isopropyl alcohol, sections were counter-stained with haematoxylin before coverslipping using an aqueous mountant.

#### Transmission electron microscopy

Cells were fixed in 2% paraformaldehyde, 2.5% glutaraldehyde in 0.1 M sodium cacodylate buffer before washing in 0.1 M sodium cacodylate buffer and post-fixing in 2% osmium tetroxide in 0.1 M sodium cacodylate buffer. Following fixation, cells were dehydrated through a graded series of alcohols and embedded in Spurrs Resin [34] according to standard electron microscopy protocol. Ultrathin sections were cut with a diamond knife (Diatome, Austria) using a Leica Ultracut S ultra-microtome (Austria) stained with both methanolic uranyl acetate and lead citrate before viewing in a transmission electron microscope, JEOL 1011 (Japan) at 60kV. Images were recorded with a MegaView III CCD cooled digital camera (Soft Imaging Systems, Münster, Germany)
